# *PTPN2* Gene Variants Are Associated with Susceptibility to Both Crohn's Disease and Ulcerative Colitis Supporting a Common Genetic Disease Background

**DOI:** 10.1371/journal.pone.0033682

**Published:** 2012-03-21

**Authors:** Jürgen Glas, Johanna Wagner, Julia Seiderer, Torsten Olszak, Martin Wetzke, Florian Beigel, Cornelia Tillack, Johannes Stallhofer, Matthias Friedrich, Christian Steib, Burkhard Göke, Thomas Ochsenkühn, Nazanin Karbalai, Julia Diegelmann, Darina Czamara, Stephan Brand

**Affiliations:** 1 Department of Medicine II - Grosshadern, Ludwig-Maximilians-University, Munich, Germany; 2 Department of Preventive Dentistry and Periodontology, Ludwig-Maximilians-University, Munich, Germany; 3 Department of Human Genetics, Rheinisch-Westfälische Technische Hochschule (RWTH), Aachen, Germany; 4 Gastrointestinal Division, Brigham & Women's Hospital, Harvard Medical School, Boston, Massachusetts, United States of America; 5 Department of Pediatrics, Hannover Medical School, Germany; 6 Max-Planck-Institute of Psychiatry, Munich, Germany; University of Tübingen, Germany

## Abstract

**Background:**

Genome-wide association studies identified *PTPN2* (protein tyrosine phosphatase, non-receptor type 2) as susceptibility gene for inflammatory bowel diseases (IBD). However, the exact role of *PTPN2* in Crohn's disease (CD) and ulcerative colitis (UC) and its phenotypic effect are unclear. We therefore performed a detailed genotype-phenotype and epistasis analysis of *PTPN2* gene variants.

**Methodology/Principal Findings:**

Genomic DNA from 2131 individuals of Caucasian origin (905 patients with CD, 318 patients with UC, and 908 healthy, unrelated controls) was analyzed for two SNPs in the *PTPN2* region (rs2542151, rs7234029) for which associations with IBD were found in previous studies in other cohorts. Our analysis revealed a significant association of *PTPN2* SNP rs2542151 with both susceptibility to CD (p = 1.95×10^−5^; OR 1.49 [1.34–1.79]) and UC (p = 3.87×10^−2^, OR 1.31 [1.02–1.68]). Moreover, *PTPN2* SNP rs7234029 demonstrated a significant association with susceptibility to CD (p = 1.30×10^−3^; OR 1.35 [1.13–1.62]) and a trend towards association with UC (p = 7.53×10^−2^; OR 1.26 [0.98–1.62]). Genotype-phenotype analysis revealed an association of *PTPN2* SNP rs7234029 with a stricturing disease phenotype (B2) in CD patients (p = 6.62×10^−3^). Epistasis analysis showed weak epistasis between the *ATG16L1* SNP rs2241879 and *PTPN2* SNP rs2542151 (p = 0.024) in CD and between *ATG16L1* SNP rs4663396 and *PTPN2* SNP rs7234029 (p = 4.68×10^−3^) in UC. There was no evidence of epistasis between *PTPN2* and *NOD2* and *PTPN2* and *IL23R*. *In silico* analysis revealed that the SNP rs7234029 modulates potentially the binding sites of several transcription factors involved in inflammation including GATA-3, NF-κB, C/EBP, and E4BP4.

**Conclusions/Significance:**

Our data confirm the association of *PTPN2* variants with susceptibility to both CD and UC, suggesting a common disease pathomechanism for these diseases. Given recent evidence that *PTPN2* regulates autophagosome formation in intestinal epithelial cells, the potential link between *PTPN2* and *ATG16L1* should be further investigated.

## Introduction

Inflammatory bowel diseases (IBD), encompassing Crohn's disease (CD) and ulcerative colitis (UC), are characterized by chronic intestinal inflammation caused by a dysregulated interaction with bacterial antigens, resulting in an exaggerated immune response in a genetically predisposed host [1], [2]. Genome-wide association studies (GWAS) have substantially improved our understanding of the molecular pathways leading to CD or UC and have so far identified almost 100 distinct genetic loci that confer IBD susceptibility including novel pathways involved in autophagy, innate immune response and proinflammatory IL-23/Th17 cell activation [3], [4], [5], [6], [7], [8], [9]. However, for many of the gene regions identified by GWAS, there is still lack of functional data and limited knowledge how these gene variants modify the IBD phenotype.

One of the recent candidate genes identified by GWAS is protein tyrosine phosphatase non-receptor type 2 (*PTPN2*), encoding the enzyme tyrosine-protein phosphatase non-receptor type 2, a member of the protein tyrosine kinases (PTP) superfamily. So far, two isoforms of PTPN2 generated from alternative splicing have been identified: a major TC45 isoform (45 kDa) containing a nuclear localization sequence and a less abundant TC48 isoform (48 kDa) anchored to the endoplasmic reticulum [10]. For TC45, various targets including Janus kinases (JAKs), signal transducer and activator of transcription (STAT) 1 and 3, p42/44 mitogen-activated protein kinase (MAPK) (extracellular signal–related kinase [ERK]), epidermal growth factor receptor (EGFR) as well as insulin receptor β (IRβ) [11], [12], [13], [14] have been identified. So far, *PTPN2* has been shown to be a susceptibility gene for celiac disease and for diabetes modifying beta-cell responses to viral RNA and apoptosis [15], [16], [17]. Recent GWAS identified *PTPN2* as susceptibility gene for CD [18], [19], [20], [21], while a GWAS meta-analyses in UC patients showed also an association with UC [22]. Interestingly, an analysis in a Dutch-Belgian cohort [23] revealed that the *PTPN2* SNP rs2542151 was only moderately CD-associated in a CD subcohort of smokers (p = 0.04), but not in the entire cohort or in the non-smoking CD cohort, implicating additional modifying factors requiring further functional analysis and replication studies.

Given the overall lack of detailed phenotype analyses of *PTPN2* in IBD, we initiated an extensive genotype-phenotype analysis in a large German cohort of IBD patients including 905 patients with CD, 318 patients with UC, and 908 healthy, unrelated controls which were genotyped for the two SNPs rs2542151 and rs7234029 in the *PTPN2* region. Based on a pathway analysis of gene relationships across implicated loci (GRAIL) of a recent GWAS meta-analysis in CD demonstrating a potential interaction between the *PTPN2*-related gene *PTPN22* and *NOD2* [19], we also performed analysis for gene-gene interaction between *PTPN2* and *NOD2* regarding CD susceptibility. Moreover, considering that *PTPN2* gene variants are - similar to *IL23R* gene variants - associated with a number of autoimmune diseases such as juvenile idiopathic arthritis [24] or type 1 diabetes [17], we also analysed for epistasis between *PTPN2* and *IL23R*. In addition, a very recent study suggests that *PTPN2* regulates autophagosome formation in intestinal epithelial cells [25]. We therefore analyzed also for potential epistasis with the CD susceptibility gene *ATG16L1*.

## Methods

### Ethics statement

Before participating in the study, all patients gave written, informed consent. In case of minors, patients' parents gave written consent. The Ethics committee of the Medical Faculty of Ludwig-Maximilians-University Munich approved this study. The study protocol was in accordance with the ethical principles for medical research involving human subjects of the Helsinki Declaration.

### Study population and genotype-phenotype analysis

Overall, 2131 individuals of Caucasian origin including 905 CD patients, 318 UC patients, and 908 healthy, unrelated controls were included in the study population. Patients with indeterminate colitis were excluded from the study. For phenotype analysis, the demographic and clinical data (behaviour and location of IBD, disease-related complications, surgical history or immunosuppressive therapy) of the patients were recorded by patient chart analysis and a detailed questionnaire including an interview at time of enrolment. The demographic characteristics of the IBD study population were collected blind to the results of the genotype analyses (Table 1). The diagnosis of CD or UC was determined according to established guidelines based on endoscopic, radiological, and histopathological criteria [26]. In CD patients, the Montreal classification based on the age at diagnosis (A), location (L), and behaviour (B) of disease [27] was used for assessment. In patients with UC, anatomic location was also based on the Montreal classification using the criteria ulcerative proctitis (E1), left-sided UC (distal UC; E2), and extensive UC (pancolitis; E3).

**Table 1:** Demographic characteristics of the IBD study population.

	Crohn's disease	Ulcerative colitis	Controls
	*n = 905*	*n = 318*	*n = 908*
**Gender**			
Male (%)	48.8	52.2	62.7
Female (%)	51.2	47.8	37.3
**Age (yrs)**			
Mean ± SD	40.9±13.3	44.2±14.8	45.8±10.3
Range	15–83	17–88	19–68
**Body mass index**			
Mean ± SD	23.0±4.2	23.9±4.5	
Range	13–41	15–54	
**Age at diagnosis (yrs)**			
Mean ± SD	26.1±12.4	28.9±14.5	
Range	1–78	2–81	
**Disease duration (yrs)**			
Mean ± SD	13.4±8.9	12.2±8.3	
Range	0–47	1–50	
**Positive family history of IBD (%)**	16.7	17.4	

### DNA extraction and genotyping of the PTPN2 variants

From all study participants, blood samples were taken and genomic DNA was isolated from peripheral blood leukocytes using the DNA blood mini kit from Qiagen (Hilden, Germany) according to the manufacturer's guidelines. The two *PTPN2* SNPs rs2542151 and rs7234029 were genotyped by PCR and melting curve analysis using a pair of fluorescence resonance energy transfer (FRET) probes in a LightCycler® 480 Instrument (Roche Diagnostics, Mannheim, Germany) as described in detail in previous studies [28], [29], [30], [31], [32], [33]. The *PTPN2* SNP rs2542151 was selected from the GWAS by Parkes et al. [18] and the Wellcome Trust Case Control Consortium (WTCCC) [20], while the rs7234029 was chosen from the study of Thompson et al. [24]. All sequences of primers and FRET probes and primer annealing temperatures used for genotyping and for sequence analysis are given in tables S1 and S2.

### Genotyping of NOD2, IL23R and ATG16L1 variants

Genotyping data of the three main CD-associated *NOD2* variants p.Arg702Trp (rs2066844), p.Gly908Arg (rs2066845), and p.Leu1007fsX1008 (rs2066847) as well as genotyping data of 10 *IL23R* SNPs (rs1004819, rs7517847, rs10489629, rs2201841, rs11465804, rs11209026 = p.Arg381Gln, rs1343151, rs10889677, rs11209032, rs1495965) were available from previous studies [28],[34], [35], [36] Nine *ATG16L1* variants (rs13412102, rs12471449, rs6431660, rs1441090, rs2289472, rs2241880 [ = p.Thr300Ala], rs2241879, rs3792106, rs4663396) have also been genotyped in a previous study [29]. For all genotyping protocols, primer and probe sequences are available on request.

### *In silico* analysis of transcription factor binding sites

We performed an *in silico* analysis for potential changes in transcription factor binding sites caused by the *PTPN2* SNPs rs2542151 and rs7234029 using the online tool TFSEARCH (http://www.cbrc.jp/research/db/TFSEARCH.html). This tool is based on the TRANSFAC database which was developed at GBF Braunschweig, Germany [37]. We used a threshold score for binding sites of 75.0 (score = 100.0 * (‘weighted sum’ - min)/(max - min); max. score = 100). For both *PTPN2* SNPs, major and minor alleles including the flanking sequences 10 bp upstream and downstream were investigated for potential changes of binding sites of human transcription factors.

### Statistical analyses

For data evaluation, we used the SPSS 13.0 software (SPSS Inc., Chicago, IL, U.S.A.) and R-2.13.1. (http://cran.r-project.org). Each genetic marker was tested for Hardy-Weinberg equilibrium in the control population. Fisher's exact test was used for comparison between categorical variables. All tests were two-tailed, considering p-values<0.05 as significant. Odds ratios were calculated for the minor allele at each SNP. Bonferroni correction was applied by calculating the threshold for statistically significant p-values as follows: p = 0.05/n, in which n gives the number of hypotheses tested. The number of tests applied (n) and the threshold for statistically significant p-values are given in the legends for all tables in which Bonferroni correction was applied. Epistasis between different SNPs was tested using the –epistasis option in PLINK (http://pngu.mgh.harvard.edu/~purcell/plink/). Haplotype based association analysis was done with PLINK using the –hap-logistic option. The two significant SNPs (rs2542151 and rs7234029) of the single-marker association study were taken into a logistic regression model for haplotype specific associations. Genotype-phenotype associations were assessed using logistic regression analysis in R.

## Results

### PTPN2 gene variants are associated with the susceptibility to both CD and UC

In all three subgroups (CD, UC, and controls), the allele frequencies of the *PTPN2* SNPs (rs2542151 and rs7234029) were in accordance with the predicted Hardy-Weinberg equilibrium (Table 2). Our analysis revealed a significant association of the *PTPN2* SNP rs2542151 with both susceptibility to CD (p = 1.95×10^−5^; OR 1.49 [1.34–1.79]) and UC (p = 3.87×10^−2^, OR 1.31 [1.02–1.68]). Moreover, the *PTPN2* SNP rs7234029 demonstrated a significant association with susceptibility to CD (p = 1.30×10^−3^; OR 1.35 [1.13–1.62]) and a trend towards association with UC (p = 7.53×10^−2^; OR 1.26 [0.98–1.62]), suggesting *PTPN2* as common susceptibility gene for both CD and UC in the German population.

**Table 2:** Associations of *PTPN2* gene markers in the CD and UC case-control cohorts.

*PTPN2* SNP	Minor allele	Crohn's disease			Ulcerative colitis			Controls
		*n = 905*			*n = 318*			*n = 908*
		MAF	p value	OR [95% CI]	MAF	p value	OR [95% CI]	MAF
rs2542151	G	0.182	1.95×10^−5^	1.49 [1.34–1.79]	0.164	3.87×10^−2^	1.31 [1.02–1.68]	0.130
rs7234029	G	0.177	1.30×10^−3^	1.35 [1.13–1.62]	0.167	7.53×10^−2^	1.26 [0.98–1.62]	0.137

Minor allele frequencies (MAF), allelic test *P*-values, and odds ratios (OR, shown for the minor allele) with 95% confidence intervals (CI) are depicted for both the CD and UC case-control cohorts.

### Haplotype analysis

Next, we analyzed haplotypes formed by the *PTPN2* SNPs rs2542151 and rs7234029 using a logistic regression model for haplotype-specific associations. The results in table 3 indicate the strongest association with CD for the TA haplotype with p = 2.37×10^−5^. There were similar results for UC with a p-value of p = 1.52×10^−2^ for the TA haplotype (Table 4).

**Table 3:** Haplotype analysis for the *PTPN2* SNPs rs2542151 and rs7234029 in the CD case-control cohort.

*PTPN2* SNP1	*PTPN2* SNP2	Haplotype	OR	95% CI	p-value
rs2542151	rs7234029	GG	1.42	1.04–1.93	1.46×10^−3^
rs2542151	rs7234029	TG	1.18	0.82–1.71	2.93×10^−1^
rs2542151	rs7234029	GA	1.45	0.92–2.32	1.88×10^−2^
rs2542151	rs7234029	TA	0.70	0.62–0.80	2.37×10^−5^

OR = odds ratio; 95% CI = 95% confidence interval.

**Table 4:** Haplotype analysis for the *PTPN2* SNPs rs2542151 and rs7234029 in the UC case-control cohort.

*PTPN2* SNP1	*PTPN2* SNP2	Haplotype	OR	95% CI	p-value
rs2542151	rs7234029	GG	1.21	0.83–1.77	0.220
rs2542151	rs7234029	TG	1.34	0.77–2.33	0.147
rs2542151	rs7234029	GA	1.37	0.76–2.47	0.151
rs2542151	rs7234029	TA	0.76	0.63–0.91	0.015

OR = odds ratio; 95% CI = 95% confidence interval.

### Genotype-phenotype analysis

Genotype-phenotype analysis (Tables 5, 6, 7, 8) revealed an association of *PTPN2* SNP rs7234029 with a stricturing disease phenotype (B2) in CD patients (p = 6.62×10^−3^; Table 6). In addition, there were weak associations of the same SNP with an early onset (A1) of CD (p = 4.25×10^−2^; Table 6). Similarly, *PTPN2* SNP rs7234029 modulates disease onset of UC (for A2: p = 3.47×10^−2^; for A3: p = 2.51×10^−2^; Table 8). In addition, we found an association of this SNP with the risk for abscess formation (p = 3.94×10^−2^) in UC (Table 8). However, none of these associations remained significant after Bonferroni correction.

**Table 5:** Genotype-phenotype associations of the *PTPN2* SNP rs2542151 in CD patients.

*PTPN2* SNP rs2542151	TT (*n = 611*)	TG (*n = 258*)	GG (*n = 36*)	P_G_	OR_G_ [95% CI]
**Age at diagnosis (yr)** *(n = 817)*					
Mean ± SD	25.32±11.91	28.06±13.48	25.19±9.61	5.96×10^−2^	0.75
Range	1–71	2–78	15–49		[0.56–1.01]
**Age at diagnosis** *(n = 817)*					
< = 16 years (A1)	126 (22.7%)	38 (16.1%)	4 (14.8%)	**4.53×10^−2^**	0.67 [0.45–0.99]
*(n = 168)*					(A1 vs. A2)
17–40 years (A2)	368 (66.4%)	161 (68.2%)	22 (81.5%)	0.285	0.79 [0.50–1.22]
*(n = 551)*					(A2 vs. A3)
>40 years (A3)	60 (10.8%)	37 (15.7%)	1 (3.7%)	**1.89×10^−2^**	0.53 [0.31–0.90]
*(n = 98)*					(A1 vs. A3)
**Location** (*n = 770)*					
Terminal ileum (L1) *(n = 113)*	73 (14.3%)	33 (14.4%)	7 (21.9%)	0.715	1.08 [0.71–1.64]
Colon (L2)	62 (12.2%)	31 (13.5%)	4 (12.5%)	0.627	1.17
*(n = 97)*					[0.72–1.74]
Ileocolon (L3)	366 (71.9%)	163 (71.2%)	21 (65.6%)	0.682	0.93
*(n = 550)*					[0.67–1.30]
Upper GI (L4)	8 (1.6%)	2 (0.9%)	0 (0%)	0.360	0.48
*(n = 10)*					[0.10–2.29]
**Behaviour** *(n = 747)*					
Non-stricturing/Non-penetrating (B1)	111 (24.3%)	49 (23.6%)	12 (42.9%)	0.645	1.08
*(n = 172)*					[0.76–1.55]
Stricturing (B2)	123 (27.0%)	57 (27.4%)	7 (25.0%)	0.941	1.01
*(n = 187)*					[0.71–1.44]
Penetrating	222 (48.7%)	102 (49.0%)	9 (32.1%)	0.729	0.95
(B3) *(n = 333)*					[0.70–1.29]
**Use of immunosuppressive agents**	no: 65 (16.6%)	35 (20.3%)	5 (22.7%)	0.237	0.77
*(n = 585)*	yes: 326 (83.4%)	137 (79.7%)	17 (77.3%)		[0.50–1.19]
**Surgery because of CD**	no: 265 (48.7%)	106 (44.7%)	19 (59.4%)	0.547	1.09
*(n = 813)*	yes: 279 (51.3%)	131 (55.3%)	13 (40.6%)		[0.82–1.47]
**Fistulas**	no: 290 (52.3%)	121 (50.2%)	18 (56.3%)	0.699	1.06
*(n = 827)*	yes: 264 (47.7%)	120 (49.8%)	14 (43.8%)		[0.79–1.42]
**Stenosis**	no: 234 (42.0%)	90 (37.5%)	16 (50.0%)	0.404	1.13
*(n = 829)*	yes: 323 (58.0%)	150 (62.5%)	16 (50.0%)		[0.84–1.53]

PG: p-value for association comparing carriers of the G-allele to individuals homozygous for T. Association results for age at diagnosis are based on median split. Uncorrected p-values<0.05 are depicted in bold. None of the p-values remained significant after Bonferroni correction for multiple testing (number of hypothesis tested: n = 15, resulting in a significance threshold of p<3.33×10^−3^).

**Table 6:** Genotype-phenotype associations of the *PTPN2* SNP rs7234029 in CD patients.

*PTPN2* SNP rs7234029	AA (*n = 612*)	AG (*n = 253*)	GG (*n = 32*)	P_G_	OR_G_ [95% CI]
**Age at diagnosis (yr)** *(n = 811)*					
Mean ± SD	25.80±12.36	26.77±12.40	25.73±12.52	0.197	0.82
Range	1–71	6–78	12–64		[0.61–1.11]
**Age at diagnosis** *(n = 811)*					
< = 16 years (A1) *(n = 168)*	124 (22.5%)	36 (15.3%)	8 (30.8%)	**4.25×10^−2^**	0.67 [0.46–0.99] (A1 vs. A2)
17–40 years (A2) *(n = 546)*	357 (64.9%)	173 (73.6%)	16 (61.5%)	0.271	1.30 [0.81–2.09] (A2 vs. A3)
>40 years (A3) *(n = 97)*	69 (12.5%)	26 (11.1%)	2 (7.7%)	0.637	0.87 [0.50–1.53] (A1 vs. A3)
**Location** (*n = 764)*					
Term. ileum (L1) (*n = 113)*	76 (14.8%)	29 (12.6%)	8 (38.1%)	0.978	0.99 [0.65–1.52]
Colon (L2) (*n = 96)*	64 (12.5%)	30 (13.0%)	2 (9.5%)	0.915	1.03 [0.65–1.61]
Ileocolon (L3) (*n = 545)*	364 (71.0%)	170 (73.9%)	11 (52.4%)	0.740	1.06 [0.76–1.48]
Upper GI (L4) (*n = 10)*	9 (1.8%)	1 (0.4%)	0 (0%)	0.157	0.22 [0.03–1.78]
**Behaviour** *(n = 686)*					
Non-stricturing -Non-penetrating (B1) *(n = 170)*	116 (25.4%)	49 (23.0%)	8 (42.1%)	0.682	0.93 [0.64–1.34]
Stricturing (B2) *(n = 187)*	110 (24.1%)	72 (33.8%)	5 (26.3%)	**6.62×10^−3^**	1.61 [1.14–2.27]
Penetrating (B3) *(n = 329)*	231 (50.5%)	92 (43.2%)	6 (31.6%)	9.06×10^−2^	0.76 [0.56–1.04]
**Use of immuno-suppressive agents** *(n = 580)*	no: 67 (17.2%)	33 (18.8%)	5 (33.3%)	0.433	0.84 [0.54–1.30]
	yes: 322 (82.8%)	143 (81.3%)	10 (66.7%)		
**Surgery because of CD** *(n = 807)*	no: 260 (47.6%)	111 (47.2%)	16 (61.5%)	0.782	0.96 [0.71–1.29]
	yes: 286 (52.4%)	124 (52.8%)	10 (38.5%)		
**Fistulas** *(n = 821)*	no: 281 (50.4%)	128 (54.2%)	18 (66.7%)	0.168	0.81 [0.61–1.09]
	yes: 277 (49.6%)	108 (45.8%)	9 (33.3%)		
**Stenosis** *(n = 823)*	no: 239 (42.8%)	82 (34.3%)	16 (61.5%)	0.111	1.28
	yes: 319 (57.2%)	157 (65.7%)	10 (38.5%)		[0.95–1.72]

PG: p-value for association comparing carriers of the G-allele to individuals homozygous for A. Association results for age at diagnosis are based on median split. Uncorrected p-values<0.05 are depicted in bold. None of the p-values remained significant after Bonferroni correction for multiple testing (number of hypothesis tested: n = 15, resulting in a significance threshold of p<3.33×10^−3^).

**Table 7:** Genotype-phenotype associations of the *PTPN2* SNP rs2542151 in UC patients.

*PTPN2* SNP rs2542151	TT (*n = 226*)	TG (*n = 80*)	GG (*n = 12*)	P_G_	OR_G_ [95% CI]
**Gender** *(n = 318)*					
Male	125 (55.3%)	36 (45.0%)	5 (41.7%)	8.03×10^−2^	1.54 [0.95–2.51]
Female	101 (44.7%)	44 (55.0%)	7 (58.3%)		
**Age at diagnosis (yrs)** *(n = 302)*					
Mean ± SD	29.45±14.60	27.95±14.13	25.08±15.54	0.253	1.34 [0.81–2.21]
Range	2–81	4–73	9–68		
**Age at diagnosis** *(n = 302)*					
< = 16 years (A1) *(n = 59)*	41 (19.1%)	16 (21.3%)	2 (16.7%)	0.926	0.97 [0.51–1.83] (A1 vs. A2)
17–40 years (A2) *(n = 183)*	126 (58.6%)	49 (65.3%)	8 (66.7%)	9.95×10^−2^	1.81 [0.89–3.67] (A2 vs. A3)
>40 years (A3) *(n = 60)*	48 (22.3%)	10 (13.3%)	2 (16.7%)	0.189	1.76 [0.76–4.07] (A1 vs. A3)
**BMI (kg/m^2^)** *(n = 209)*					
Mean ± SD	23.92±4.74	23.87±3.83	23.67±4.71	0.995	1.00 [0.55–1.82]
Range	15–54	16–36	15–30		
**Location** *(n = 200)*					
Proctitis (E1) *(n = 24)*	15 (12.0%)	7 (10.6%)	2 (22.2%)	0.329	1.55 [0.64–3.70]
Left-sided UC (E2) *(n = 96)*	73 (58.4%)	20 (30.3%)	3 (33.3%)	0.184	0.68 [0.38–1.20]
Extensive UC (E3) *(n = 80)*	37 (29.6%)	39 (59.1%)	4 (44.4%)	0.473	1.22 [0.83–1.80]
**Extra-intestinal manifestations** *(n = 191)*	no: 87 (64.4%)	33 (68.8%)	5 (62.5%)	0.652	0.86 [0.44–1.66]
	yes:48 (35.6%)	15 (31.3%)	3 (37.5%)		
**Use of immuno-suppressive agents**	no: 50 (26.0%)	14 (20.9%)	2 (22.2%)	0.394	1.32 [0.70–2.50]
*(n = 268)*	yes: 142 (74.0%)	53 (79.1%)	7 (77.8%)		
**Abscesses** *(n = 240)*	no: 160 (96.4%)	61 (93.8%)	7 (77.8%)	0.151	2.35 [0.73–7.56]
	yes: 6 (3.6%)	4 (6.2%)	2 (22.2%)		

PG: p-value for association comparing carriers of the G-allele to individuals homozygous for A. Association results for age at diagnosis and BMI are based on median split. None of the p-values remained significant after Bonferroni correction for multiple testing (number of hypothesis tested: n = 12, resulting in a significance threshold of p<4.167×10^−3^).

**Table 8:** Genotype-phenotype associations of the *PTPN2* SNP rs7234029 in UC patients.

*PTPN2* SNP rs7234029	AA *(n = 220)*	AG *(n = 83)*	GG *(n = 11)*	P_G_	OR_G_ [95% CI]
**Gender** *(n = 314)*					
Male	119 (54.1%)	42 (50.6%)	5 (45.5%)	0.506	1.18 [0.73–1.91]
Female	101 (45.9%)	41 (49.4%)	6 (54.5%)		
**Age at diagnosis (yrs)** *(n = 298)*					
Mean ± SD	29.95±14.90	26.32±13.49	30.50±12.70	0.401	1.24 [0.75–2.05]
Range	3–81	2–73	14–57		
**Age at diagnosis** *(n = 298)*					
<16 years (A1) *(n = 57)*	37 (17.5%)	19 (25.0%)	1 (10.0%)	0.558	1.21 [0.64–2.26] (A1 vs. A2)
17–40 years (A2) *(n = 181)*	125 (59.0%)	49 (64.5%)	7 (70.0%)	**3.47×10^−2^**	2.24 [1.06–4.73] (A2 vs. A3)
>40 years (A3) *(n = 60)*	50 (23.6%)	8 (10.5%)	2 (20.0%)	**2.51×10^−2^**	2.70 [1.13–6.45] (A1 vs. A3)
**BMI (kg/m^2^)** *(n = 207)*					
Mean ± SD	23.97±4.66	24.10±4.26	22.90±3.18	0.975	0.99 [0.54–1.82]
Range	15–54	15–36	20–29		
**Location** *(n = 258)*					
Proctitis (E1) *(n = 24)*	13 (7.2%)	11 (16.4%)	0 (0%)	7.75×10^−2^	2.15 [0.92–5.05]
Left-sided UC (E2) *(n = 96)*	71 (39.2%)	20 (29.9%)	5 (50.0%)	0.305	0.74 [0.42–1.31]
Extensive UC (E3) *(n = 138)*	97 (53.6%)	36 (53.7%)	5 (50.0%)	0.960	0.99 [0.58–1.68]
**Extra-intestinal manifestations** *(n = 188)*	no: 87 (66.4%)	33 (63.5%)	3 (60.0%)	0.666	1.15 [0.60–2.21]
	yes: 44 (33.6%)	19 (36.5%)	2 (40.0%)		
**Use of immunosuppressive agents** *(n = 266)*	no: 50 (26.5%)	15 (22.4%)	1 (10.0%)	0.332	1.37 [0.72–2.60]
	yes: 139 (73.5%)	52 (77.6%)	9 (90.0%)		
**Abscesses** *(n = 238)*	no: 161 (97.0%)	58 (92.1%)	7 (77.8%)	**3.94×10^−2^**	3.47 [1.06–11.32]
	yes: 5 (3.0%)	5 (7.9%)	2 (22.2%)		

PG: p-value for association comparing carriers of the G-allele to individuals homozygous for A. Association results for age at diagnosis and BMI are based on median split. Uncorrected p-values<0.05 are depicted in bold. None of the p-values remained significant after Bonferroni correction for multiple testing (number of hypothesis tested: n = 12, resulting in a significance threshold of p<4.167×10^−3^).

### Analysis for epistasis between PTPN2 and the main CD susceptibility genes NOD2, IL23R and ATG16L1

In addition, we analyzed for potential epistasis between *PTPN2* and the three main CD susceptibility genes *NOD2, IL23R* and *ATG16L1*, given recent evidence for a potential functional interaction between these genes. For example, GRAIL analysis identified a link between the *PTPN2*-related gene *PTPN22* and *NOD2* [19]. In addition, *PTPN2* regulates autophagosome formation in human intestinal epithelials cells, suggesting a potential link to *ATG16L1* [25]. Epistasis analysis demonstrated weak epistasis between the *ATG16L1* SNP rs2241879 and *PTPN2* SNP rs2542151 (p = 0.024) in the CD cohort (Table 9) and between *ATG16L1* SNP rs4663396 and *PTPN2* SNP rs7234029 (p = 4.68×10^−3^) in the UC cohort (Table 10). However, significance of these associations was lost after correcting for multiple testing (Bonferroni correction). In addition, there was no evidence for epistasis between *PTPN2* and CD-associated variants in the *NOD2* and *IL23R* genes.

**Table 9:** Analysis for epistasis between *PTPN2* SNPs and gene markers located in *NOD2*, *IL23R* and *ATG16L1* in the CD-case control population.

Epistasis between	*PTPN2* SNP rs2542151	*PTPN2* SNP rs7234029
***NOD2*** ** SNPs**		
rs2066844 (p.Arg702Trp)	0.607	0.498
rs2066845 (p.Gly908Arg	0.219	0.916
rs2066847(p.Leu1007fsX1008)	0.208	0.276
***IL23R*** ** SNPs**		
rs1004819	0.556	0.244
rs7517847	0.723	0.916
rs10489629	0.395	0.642
rs2201841	0.303	0.414
rs11465804	0.485	0.887
rs11209026 (p.Arg381Gln)	0.943	0.754
rs1343151	0.277	0.978
rs10889677	0.508	0.417
rs11209032	0.213	0.290
rs1495965	9.86×10^−2^	0.258
***ATG16L1*** ** SNPs**		
rs13412102	0.620	0.358
rs12471449	0.419	0.383
rs6431660	5.73×10^−2^	0.394
rs1441090	0.389	0.437
rs2289472	0.102	0.404
rs2241880 (p.Thr300Ala)	8.07×10^−2^	0.570
rs2241879	**2.37×10^−2^**	0.382
rs3792106	0.303	0.930
rs4663396	9.76×10^−2^	0.109

Uncorrected p-values<0.05 are depicted in bold. None of the p-values remained significant after Bonferroni correction for multiple testing (number of hypothesis tested: n = 44, resulting in a significance threshold of p<1.136×10^−3^).

**Table 10:** Epistasis between *PTPN2* SNPs and gene markers located in *NOD2, IL23R* and *ATG16L1* in the UC-case control population.

Epistasis between	*PTPN2* SNP rs2542151	*PTPN2* SNP rs7234029
***NOD2*** ** SNPs**		
rs2066844 (p.Arg702Trp)	0.611	0.219
rs2066845 (p.Gly908Arg	0.385	0.555
rs2066847(p.Leu1007fsX1008)	0.137	0.522
***IL23R*** ** SNPs**		
rs1004819	0.869	0.425
rs7517847	0.561	0.972
rs10489629	0.177	0.844
rs2201841	0.711	0.421
rs11465804	0.465	0.265
rs11209026 (p.Arg381Gln)	0.471	0.831
rs1343151	0.525	0.331
rs10889677	0.889	0.303
rs11209032	0.649	0.330
rs1495965	0.847	0.740
***ATG16L1*** ** SNPs**		
rs13412102	0.553	0.749
rs12471449	0.762	8.21×10^−2^
rs6431660	0.298	0.104
rs1441090	0.455	0.544
rs2289472	0.392	0.100
rs2241880 (p.Thr300Ala)	0.536	0.615
rs2241879	0.345	0.423
rs3792106	0.787	0.714
rs4663396	0.217	**4.68×10^−3^**

Uncorrected p-values<0.05 are depicted in bold. None of the p-values remained significant after Bonferroni correction for multiple testing (number of hypothesis tested: n = 44, resulting in a significance threshold of p<1.136×10^−3^).

### *In silico* analysis of PTPN2 SNPs identifies differences in potential transcription factor binding sites caused by SNP rs7234029

Finally, we investigated if the two *PTPN2* SNPs (including the surrounding sequences as detailed in the Methods section) result in changes of transcription factor binding sites. This *in silico* analysis demonstrated for SNP rs7234029 differences between major and minor allele regarding the binding probability of several transcription factors including GATA-1, GATA-2, GATA-3, HSF2, NF-κB, C/EBP, E4BP4, SREBP, and HLF. While the transcription factors GATA-1, GATA-2, GATA-3 and HSF2 were predicted to bind with very high probability to the sequence comprising the major A allele, predicted binding to the minor G allele was substantially lower (Table 11). In contrast, the binding score for the transcription factors NF- κB, C/EBP, E4BP4, SREBP, and HLF were higher for the minor G allele. The details of this analysis are shown in table 11. In contrast, no major changes regarding transcription factor binding sites were found for SNP rs2542151 which is located approximately 5.5 kb downstream of *PTPN2* (data not shown).

**Table 11:** Potential transcription factor binding sites in the genomic region harboring the *PTPN2* SNP rs7234029.

Transcription factor	Binding score major allele (A)	Binding score minor allele (G)	Consensus sequence	Position relative to SNP
**GATA-X**	**95.2**	**80.1**	NGATAAGNMNN	−2 to +8
**GATA-1**	**94.8**	**80.2**	NNCWGATARNNNN	−5 to +7
**GATA-2**	**85.8**	**65.8**	NNNGATRNNN	−4 to +5
**GATA-3**	**83.4**	**64.1**	NNGATARNG	−3 to +5
**HSF2**	75.0	62.2	NGAANNWTCK	−3 to +6
**NF-κB**	**68.5**	**79.4**	GGGAMTTYCC	−1 to +8
**C/EBP**	69.5	78.5	NGWNTKNKGYAAKNSAYA	−8 to +9
**E4BP4**	**65.7**	**76.0**	NRTTAYGTAAYN	−6 to +5
**SREBP**	67.2	75.0	NATCACGTGAY	−6 to +4
**HLF**	66.9	75.0	RTTACTYAAT	−5 to +4

The potential transcription factor binding sites were analyzed *in silico* with the program TFSEARCH (http://www.cbrc.jp/research/db/TFSEARCH.html.). Only binding sites with binding score differing more that 5 points between the two alleles are presented. Scores differing more than 10 points are depicted in bold. The binding score threshold for each allele was set to 75.0.

Nucleotide codes: K = G or T, M = A or C, R = A or G, S = C or G, W = A or T, N = A, G, C or T.

## Discussion

Our detailed analysis of a large IBD cohort demonstrates that *PTPN2* is a common susceptibility gene for both CD and UC, adding *PTPN2* to the growing list of common susceptibility genes of CD and UC. So far, 99 IBD susceptibility genes have been identified (n = 71 in CD and n = 47 in UC) [19], [22]. At least 28 susceptibility loci, including *PTPN2*, are shared between CD and UC [19], [22]. Our results confirm previous studies in which *PTPN2* has been shown to be associated with CD [18], [19], [20], [21]. A very recent meta-analysis of UC susceptibility genes by Anderson et al. reported also an association of *PTPN2* (rs1893217) with UC [22], suggesting that *PTPN2* is a susceptibility gene for both UC and CD which is in complete agreement with the results of our study. The *PTPN2* variant rs1893217, which has been shown to be associated with IBD in several studies [19], [22], is in complete linkage disequilibrium with the *PTPN2* SNP rs2542151, which was investigated in our study. In a smaller analysis from New Zealand, an association of *PTPN2* with CD but not of *PTPN22* could be shown [38]. Studies in an Italian cohort [39] and in a Dutch-Belgian cohort [40] also reported *PTPN2* to be a susceptibility gene for CD.

In addition, we performed a detailed genotype-phenotype analysis. Genotype-phenotype analysis revealed an association of *PTPN2* SNP rs7234029 with a stricturing disease phenotype in CD patients. In addition, we found evidence for weak associations of rs7234029 and rs2542151 with the age of IBD onset. However, after Bonferroni correction, most of these associations lost significance arguing against a strong disease-modifying role for *PTPN2* such as shown for *NOD2*. Considering that *PTPN2* predisposes to both CD and UC, someone may hypothesize that it would be associated with a predominant colonic disease location; however, we were unable to show such an association in our detailed genotype-phenotype analysis.

Moreover, we performed epistasis analysis investigating potential gene-gene interactions between *PTPN2* and the three main CD susceptibility genes *NOD2, IL23R* and *ATG16L1*. A recent GWAS meta-analysis demonstrated for these three genes the strongest association of all 71 identified CD risk genes with CD susceptibility [19]. However, there was no epistasis between *PTPN2* and *IL23R*, although both genes predispose to autoimmune diseases. For example, associations of *IL23R* could be shown for CD and UC [3], [19], psoriasis [41] and ankylosing spondylitis [42]. *PTPN2* is associated with juvenile idiopathic arthritis [24], rheumatoid arthritis, celiac disease [19], [43], type 1 diabetes [20], [44] and Graves' disease [44], providing an explanation for the increased incidence of several of these diseases in IBD patients.

In contrast, epistasis analysis demonstrated evidence for weak epistasis between the *ATG16L1* SNP rs2241879 and *PTPN2* SNP rs2542151 (p = 0.024) in the CD cohort and between *ATG16L1* SNP rs4663396 and *PTPN2* SNP rs7234029 (p = 4.68×10^−3^) in the UC cohort, which, however, was lost after Bonferroni correction. Previous studies, including work from our own group [29], indicate that autophagy genes such as *ATG16L1* and *IRGM* play an important role in CD susceptibility and not UC susceptibility. Given the epistasis between the *ATG16L1* SNP rs4663396 and the *PTPN2* SNP rs7234029 in the UC cohort, our study suggests that autophagy genes may have – in combination with “true” UC susceptibility genes such as *PTPN2* - also a role in UC susceptibility. However, the rather weak epistasis between these two genes needs further confirmation in large replication studies.

The potential epistasis between *PTPN2* and *ATG16L1* would be highly interesting, given very recent evidence that PTPN2 regulates autophagosome formation in human intestinal epithelial cells [25]. Scharl *et al.* showed that knockdown of *PTPN2* causes impaired autophagosome formation and dysfunctional autophagy [25]. This resulted in increased levels of intracellular *Listeria monocytogenes* and enhanced apoptosis of intestinal epithelial cells in response to TNF-α and IFN-γ [25]. Similar results were found in primary colonic lamina propria fibroblasts isolated from CD patients who were carriers of the CD-associated *PTPN2* SNP rs2542151 [25] which was the most strongly CD-associated SNP in our study. In the study by Scharl *et al.*, presence of the CD-associated *ATG16L1* SNP rs2241880 prevented the TNF-α/IFN-γ-mediated increase in PTPN2 protein expression which resulted in impaired autophagosome formation [25]. Interestingly, intestinal biopsies from CD patients with either CD-associated *ATG16L1* or *PTPN2* SNPs showed aberrant expression patterns of LC3B, a marker for autophagic membranes [25]. Scharl *et al.* therefore hypothesized that the combined dysfunction of the CD susceptibility genes *PTPN2* and *ATG16L1* may contribute to the pathogenesis of CD [25]. Our results demonstrating epistasis between CD-associated *PTPN2* and *ATG16L1* gene variants support this hypothesis. In addition, it has been shown that *PTPN2* regulates muramyl dipeptide (MDP)-induced autophagosome formation [45]. These experiments also demonstrated that the CD-associated *PTPN2* variant rs1893217 impairs autophagy [45]. Given the physical interaction of ATG16L1 and the MDP receptor NOD2 during autophagy [46], CD-associated *PTPN2* variants may increase the CD risk by interfering with ATG16L1-/NOD2-mediated autophagy. In addition, GRAIL analysis identified a link between the *PTPN2*-related gene *PTPN22* and *NOD2* [19]. However, we were unable to demonstrate epistasis between *PTPN2* and the three main CD-associated *NOD2* variants p.Arg702Trp (rs2066844), p.Gly908Arg (rs2066845), and p.Leu1007fsX1008 (rs2066847) on a genetic level. Additional studies suggest that PTPN2 plays an overall protective role in the intestine, particularly by limiting IFN-γ-induced signaling and consequent barrier defects [47] as well as by modulating TNF-α responses [48].

To further elucidate the potential functional consequences by which *PTPN2* SNPs modulate IBD susceptibility, we performed an *in silico* analysis regarding potential changes in binding sites for transcription factors. This analysis revealed that the SNP rs7234029 modulates potentially the binding sites of several transcription factors including GATA-3, NF-κB, C/EBP, and E4BP4 which were all shown to be involved in inflammatory processes. GATA-3 is a major transcription factor involved in differentiation of Th2 cells [49] which play a fundamental role in the pathogenesis of UC. NF-κB up-regulates the gene expression of many proinflammatory cytokines including IL-12 [50]. Together with NF-κB, C/EBP is activated by signaling via pattern recognition receptors (PRRs) which respond to pathogen-associated molecular patterns (PAMPs) or host-derived damage-associated molecular patterns (DAMPs). Both transcription factors play therefore a pivotal role in inflammatory disorders. E4BP4 is essential for the development of natural killer (NK) cells and CD8α+ conventional dendritic cells; it plays also a role in macrophage activation, polarisation of CD4+ T cell responses and B cell class switching to IgE [51]. Interestingly, E4BP4 may also modulate IL-12 expression [52] which plays a key role in the pathogenesis of CD. The predicted binding of the transcription factors NF-κB, C/EBP and E4BP4 was stronger to the CD-associated minor allele of SNP rs7234029 than to the protective major allele, suggesting that the increased CD risk may be partially modulated via the stronger activation of these proinflammatory transcription factors.

In summary, we confirm *PTPN2* as common susceptibility gene for CD and UC. Genotype-phenotype analysis could not identify a clear phenotype associated with these variants. A potential association of *PTPN2* SNP rs7234029 with a stricturing disease phenotype in CD patients (p = 6.62×10^−3^) needs further confirmation in larger cohorts or meta-analyses which are currently organized by the subphenotyping committee of the International IBD Genetics Consortium. Our *in silico* analysis predicted that the increased CD risk mediated by rs7234029 may be related to a stronger activation of proinflammatory transcription factors such as NF-κB, C/EBP and E4BP4. This study revealed a potential interaction between *PTPN2* and *ATG16L1* regarding susceptibility of CD and UC. However, given the rather weak interaction, this has to be further investigated. Interestingly, this finding supports the results of a very recent functional study demonstrating a major role for PTPN2 in the autophagosome formation in human intestinal epithelial cells [25]. This suggests that different IBD-related pathways may converge in common functional “endpoints” such as autophagy resulting in increased IBD susceptibility in affected patients.

## Supporting Information

Table S1 **Primer sequences (F: forward primer, R: reverse Primer), FRET probe sequences, and primer annealing temperatures used for genotyping of** ***PTPN2*** **variants.** Note: FL: Fluorescein, LC610: LightCycler-Red 610; LC640: LightCycler-Red 640. The polymorphic position within the sensor probe is underlined. A phosphate is linked to the 3′-end of the acceptor probe to prevent elongation by the DNA polymerase in the PCR.
(DOC)

Table S2 **Primer sequences used for the sequence analysis of the** ***PTPN2*** **variants.**
(DOC)
