# Supplementary material for: PTPN2 Gene Variants Are Associated with Susceptibility to Both Crohn's Disease and Ulcerative Colitis Supporting a Common Genetic Disease Background
Source: PLoS One. 2012 Mar 21;7(3):e33682. doi: 10.1371/journal.pone.0033682 (PMC3310077; doi:10.1371/journal.pone.0033682)
Supplement: Table S1 — Primer sequences (F: forward primer, R: reverse Primer), FRET probe sequences, and primer annealing temperatures used for genotyping of PTPN2 variants. Note: FL: Fluorescein, LC610: LightCycler-Red 610; LC640: LightCycler-Red 640. The polymorphic position within the sensor probe is underlined. A phosphate is linked to the 3′-end of the acceptor probe to prevent elongation by the DNA polymerase in the PCR. (DOC) [file pone.0033682.s001.doc]

**Supplemental Table S1**

| **Polymorphism** | **Annealing temperature** | **Primer sequences** | **FRET probe sequences** |
| --- | --- | --- | --- |
| rs7234029 | 60°C | F: AgCACTACAggTAgTCACATgggTA  R: CCTTTTAAAATgTCAgCACCTTAg | gTTgTgACTTACCTgATgTTACAAT -FL  LC640- CTgCTAgTgTCATAACTgggACTCAAATCC |
| rs2542151 | 60°C | F: gATgCCACgTgggCgCTgT  R: gAgCgAAgTCCCTATCgCA | TCTCAggAAgCgCCCgA -FL  LC610- AAggCATTggCgAAgTggggCTAC |

**Supplemental Table S1.** Primer sequences (F: forward primer, R: reverse Primer), FRET probe sequences, and primer annealing temperatures used for genotyping of *PTPN2* variants. Note: FL: Fluorescein, LC610: LightCycler-Red 610; LC640: LightCycler-Red 640. The polymorphic position within the sensor probe is underlined. A phosphate is linked to the 3'-end of the acceptor probe to prevent elongation by the DNA polymerase in the PCR.
