# Supplementary material for: PTPN2 Gene Variants Are Associated with Susceptibility to Both Crohn's Disease and Ulcerative Colitis Supporting a Common Genetic Disease Background
Source: PLoS One. 2012 Mar 21;7(3):e33682. doi: 10.1371/journal.pone.0033682 (PMC3310077; doi:10.1371/journal.pone.0033682)
Supplement: Table S2 — Primer sequences used for the sequence analysis of the PTPN2 variants. (DOC) [file pone.0033682.s002.doc]

**Supplemental Table S2**

| **Polymorphism** | **Primer sequences** |
| --- | --- |
| rs7234029 | AgCACTACAggTAgTCACATgggTA  CCTTTTAAAATgTCAgCACCTTAg |
| rs2542151 | CCTgCTCCTgTCTCCCAAA  TACACggTggAAgACCATTAC |

**Supplemental Table S2.** Primer sequences used for the sequence analysis of the *PTPN2* variants.
